# Evaluation of the oral health-related quality of life in patients with odontogenic fascial space abscesses and underlying conditions – a prospective questionnaire-based study

**DOI:** 10.1186/s13005-019-0201-0

**Published:** 2019-06-21

**Authors:** Lara Schorn, Felix Schrader, Rita Depprich, Julian Lommen, Norbert R. Kübler, Henrik Holtmann

**Affiliations:** 1 Department of Oral-, Maxillo- and Plastic Facial Surgery, Heinrich-Heine-University Duesseldorf, Moorenstr. 5, 40225 Duesseldorf, Germany; 2 Department of Oral, Maxillo- and Plastic Facial Surgery, Malteser Krankenhaus St. Johannes-Stift, Johannisstraße 21, 47198 Duisburg, Germany

**Keywords:** Oral health-related quality of life, OHIP, OHIP-G, Odontogenic abscess, Fascial space abscess

## Abstract

**Background:**

Oral Health-Related Quality of Life (OHRQoL) is an important aspect of quality of life. Acute illnesses, as well as chronic diseases, can have a strong, persisting impact on an individual’s quality of life. This study evaluates OHRQoL of patients with odontogenic fascial space abscesses, the underlying conditions, and its consequences for clinical routines.

**Methods:**

The research group consisted of patients presenting themselves to the emergency room or elective clinic of the Department for Cranio-Maxillofacial and Plastic Surgery (*n* = 94). The validated German version of the Oral Health Impact Profile (OHIP-G) and additional questions (including habits and routines in oral hygiene) with an anamnestic recall period of 1 month was used to evaluate OHRQoL shortly after emergency treatment (baseline) and again after 3–6 months’ follow-up. Ninety-four patients completed the questionnaire at baseline, 54 completed both questionnaires. Additionally, OHIP-G scores were compared to those of the non-impacted general German population.

**Results:**

Results showed a significant difference in OHIP-G scores from baseline to follow-up (*p* = 0.001). Overall a mean of 55.24 (±37.02) points was scored at baseline and a mean of 37.02 (±35.79) points was scored at follow-up. Patients scored higher than participants of a representative study of the general German population.

**Conclusion:**

Overall results suggest an increase in OHRQoL 3–6 months after acute treatment. Nevertheless, OHRQoL of patients suffering from odontogenic fascial space abscesses seems to remain generally lower than the OHRQoL of the general German population.

**Trial registration:**

Trial registration: Central Study Register of the University Hospital Duesseldorf, Registration-ID: 2016085405. Registered 24 August 2016.

## Background

The World Health Organization defines quality of life as “an individual’s perception of their position in life in the context of the culture and value systems in which they live and in relation to their goals, expectations, standards and concerns” (http://www.who.int/healthinfo/survey/whoqol-qualityoflife/en/). Oral Health-Related Quality of Life (OHRQoL) as a part of quality of life is a constant topic in maxillofacial research [1]. Tools like the dental impact profile or the General Oral Health Assessment Index (GOHAI) have been developed in order to make OHRQoL epidemiologically comparable [2, 3]. The most common instrument is the Oral Health Impact Profile (OHIP). The OHIP-49 was introduced by Slade and Spencer in 1994 [4]. Since then the OHIP has been further developed and was translated into several languages including German (OHIP-G), Japanese (OHIP-J), Hungarian (OHIP-H), and Swedish (OHIP-S), and has eventually been adjusted to country-specific conditions [5–8]. The OHIP-49 consists of 49 items representing seven subsets: functional limitation, physical pain, psychological discomfort, physical disability, psychological disability, social disability, and handicap. In addition to the English version of the OHIP in the German version four more items have been added that belong to neither subcategory. Answers were given on a 5-point unipolar ordinal scale (0 = “never”, 1 = “hardly ever”, 2 = “occasionally”, 3 = “fairly often”, and 4 = “very often”). OHRQoL is evaluated by the sum of all 49 OHIP items with potential summary scores ranging from 0 to 196 points. A high score indicates heavier or more problems resulting in a lower OHRQoL. There have been numerous studies using the OHIP-49 mainly concentrating on chronic facial diseases including temporomandibular joint disorders (TMJ), trigeminal neuralgia or Medication-Related Osteonecrosis of the Jaw (MRONJ) [9–11]. However, acute conditions can have long lasting effects on the OHRQoL of patients as well [10].

A fairly common and at the same time simple but severe enough acute disease to evaluate OHRQoL in maxillofacial patients are odontogenic fascial space abscesses. Odontogenic fascial space abscesses often present as mixed infections [12]. Reasons for the development of odontogenic abscesses can be pulpitis, caries, insufficient root canal fillings, trauma, periodontitis or pericoronitis [13]. Without treatment, bacteria might spread throughout the bone leading to acute osteomyelitis. Further distribution leads to periostitis and cellulitis of the surrounding tissues eventually spreading to fascial spaces resulting in acute respiratory distress, and difficulties in swallowing [14]. When the abscess extends to involve the mediastinum or peritonsillar spaces patients present with a mortality rate of up to 40% regardless of aggressive antibiotic therapy, debridement or intensive care unit supervision [15]. Apart from small paramandibular abscesses, palatinal abscesses or abscesses of the canine fossa, which are incised intraorally, patients are usually hospitalised, and incision and drainage is performed extraorally under general anesthesia using two drains as a minimum [16, 17]. This cannot only lead to lasting functional and aesthetic disorders but also to prolonged psychological stress and discomfort.

This study evaluates the impact of odontogenic fascial space abscesses and its treatment onto the oral health-related quality of life during hospitalisation and 3–6 months after. Furthermore, it tries to determine underlying conditions not only for abscess formation but also for patients’ personal liability or resilience towards psychological distress determined by oral problems.

## Methods

In this prospective clinical study, the OHRQoL of patients suffering from odontogenic abscesses spreading to fascial spaces was determined. The research group consisted of patients presenting themselves or being transferred by resident doctors or dentists to the emergency room or elective clinic of the Department for Cranio-Maxillofacial and Plastic Surgery of the University Hospital Duesseldorf. The Ethics-Committee of the Heinrich-Heine University of Duesseldorf granted approval for the study. In order to obtain significant data the ideal sample size was calculated using G*Power Version 3.1. (2014) (Heinrich-Heine-University Duesseldorf, Germany) and was set to a number of at least 54 patients completing both questionnaires.

Inclusion criteria were diagnosis of odontogenic abscess leading to severe inflammation of the fascial spaces, age 18–90 years, the ability to read and understand German, and informed written consent. Diagnostic criteria for abscesses or severe inflammation were difficulties in swallowing, restricted movement or locking of the jaw, fever, respiratory distress. The abscess or inflammation had to be located in fascial spaces of the head and neck and be of odontogenic origin. Exclusion criteria were lack of or inability of informed consent and participants with a major systemic illness leading to altered pain sensitivity and patients suffering from other pain-related orofacial illnesses such as TMJ, MRONJ, etc.

**Fig. 1:**
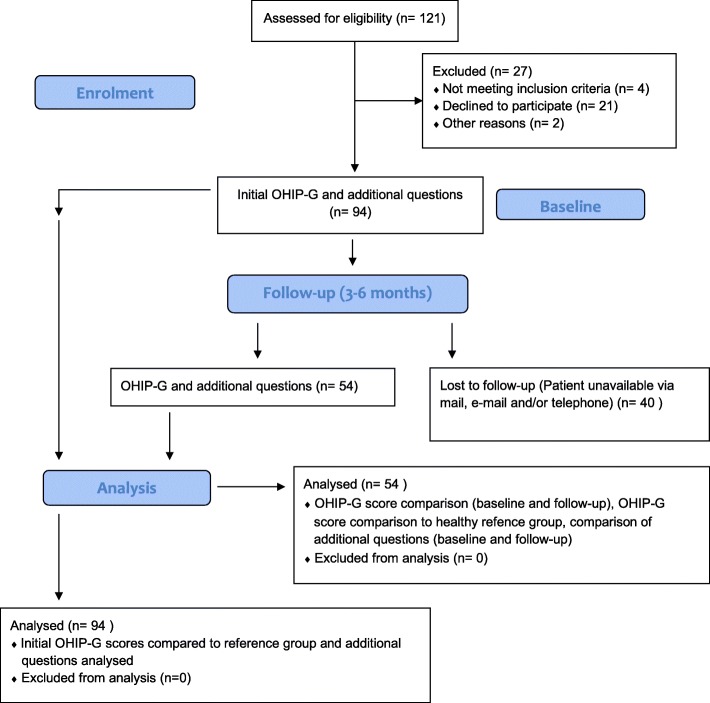
CONSORT - Flow Chart. Study design

Over a period of 2 years, 121 patients met the fundamental inclusion criteria. One hundred nine patients had to be admitted for intravenous antibiotics and/or incision and drainage. A total of 94 participants agreed to take part in this study. Written informed consent was obtained from all individual participants included in the study. The participants answered the validated German version of the Oral Health Impact Profile (see explanation below) and additional questions with an anamnestic recall period of 1 month before, during or shortly after emergency treatment (baseline). A standardised explanation of the questionnaires was used [5]. After 3–6 months patients were asked to answer the OHIP-G and further questions again (follow-up). The questionnaire was self-completed by all participants. Special attention was laid upon completion of the entire OHIP-G. Answers for additional items could be left out. Ninety-four patients completed the first survey, 54 of those completed both (Fig. 1). According to comparable studies the minimally important difference being considered clinically significant is a 6-point difference [18]. The additional four items suggested in the German version of the OHIP-49 “OHIP-53” or “OHIP-G” [5] were added and evaluated the same way. OHIP-G scores at initial consultation were compared to those after 3–6 months. No difference in OHRQoL at baseline in comparison to follow-up was assumed (null hypotheses). Additionally OHIP-G scores were compared to those of the non-impacted general German population [19]. Further questions regarding habits and oral hygiene were set in single choice mode (for the questions see Table 2). OHIP-G scores at initial consultation were compared to those after 3–6 months.

Data analysis was performed using IBM SPSS statistics for Mac version 26 (SPSS Inc., Chicago, IL, USA) and Microsoft Excel for Mac Version 16.16.3. Means and standard deviations were evaluated (mean ± SD). Primarily the simple sum of the 49 items was used for comparison. Secondarily the sums of the seven subsets were compared. Although the OHIP-Scores showed no normal distribution a T-test for paired samples was used to detect significant differences in total scores in order to evaluate the range of difference as well. For the sub categories and single items the Wilcoxon Matched Pairs test was used to detect significant differences. Additionally, percentages of the total scores were compared to scores for the unaffected German population assessed by John and Micheelis in 2003 [19]. Hereby percentile ranks of the published data were compared and tested for significance using a Chi-square test. For the additional items values and frequencies were described and compared by the use of either the McNemar-Browker test or the Wilcoxon test. The level of significance was set to *p* = < 0.05. *P* = < 0.01 was considered to represent highly significant differences.

## Results

### OHIP-G scores

**Fig. 2:**
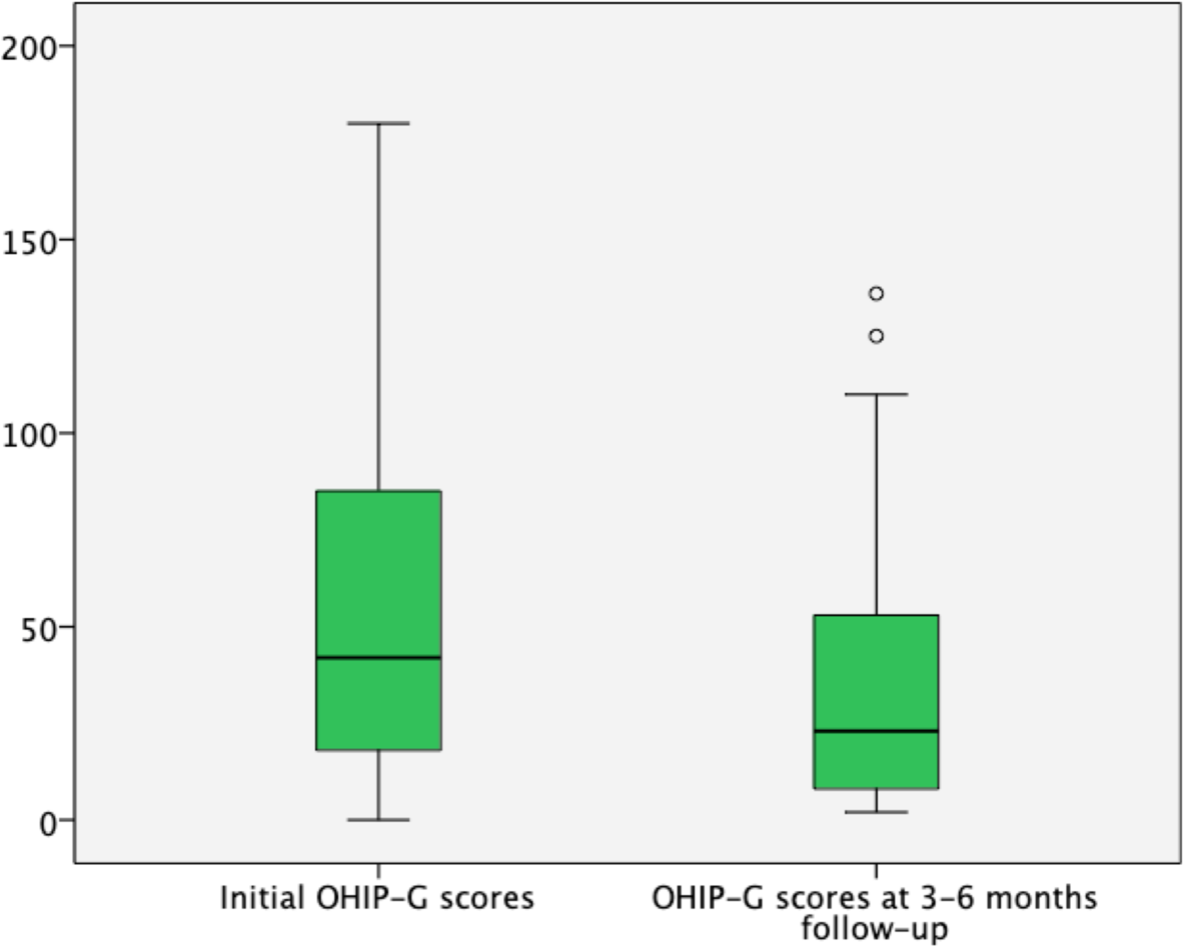
Box-Plot diagrams of total OHIP-G scores for patients with fascial space abscesses

Patients’ age ranged from 20 to 89 years. Initially, total OHIP-G scores ranged from 0 to 180 points (*n* = 94). The mean score was 55.24 (±37.02). At follow-up scores raged from 2 to 136 points (*n* = 54). The mean score was 37.02 (±35.79). A significant difference between baseline and follow-up could be assessed (*p* = 0.001) (Fig. 2). Furthermore significant differences showed in nearly every subset except for “social disability”, and 24 of the single items showed significantly different means at follow-up than at baseline.

On average womens’ results were higher than mens’ (initial womens’ mean: 74.45 (±45.023), initial mens’ mean 39.73 (±32.004), follow-up womens’ mean 44,77 (±37.382), follow-up mens’ mean 31.69 (±34.230)). In a representative study of the general German population, evaluated by John et al. in 2003, 80% of the participants without dentures scored lower than 22 points. OHIP-G scores of patients wearing dentures in this comparative study were higher [19]. With an initial mean of 57.36 (±44.35) (for patients without dentures) and a 95% confidence interval of 45.93–69.35, patients with fascial space abscesses scored significantly higher in our study. At follow-up OHIP-G scores with a mean of 30.80 (for patients without dentures) and a 95% confidence interval of 19.07 and 42.53 patients’ scores were still distinctly higher than the unaffected German population without dentures. Patients wearing dentures initially reached 50.96 (±39.22, CI 34.40–67.52) points and 44.79 (±39.92, CI 27.93–61.65) at follow-up.

### Additional questions

All 94 patients were asked additional questions to evaluate underlying reasons for the abscess formation, 54 answered additional follow-up questions as well.

Before hospitalisation 21.3% evaluated their own oral health status at baseline as “bad”, 53.2% found it to be “average”, 21.3% regarded their oral health status as “good”, and 4.3% as “very good”. At follow-up 19.6% evaluated their own oral health status at baseline as “bad”, 47.1% found it to be “average”, 29.4% regarded their oral health status as “good”, and 3.9% as “very good”. Initially 70.3% said to brush their teeth at least twice a day, 2.1% never brushed their teeth. 97.8% used a toothbrush and 95.7% a toothpaste for oral hygiene. 63% visited a dentist regularly and 48.1% said to consult a dentist at least once a year, whereas 38.3% do not regularly do dental check-ups. 93.3% planned to see a dentist more regularly in the future. 6.4% claimed that their last dental consultation was longer than 5 years ago. With 73.3% the main reason for emergency admittance was extensive swelling. 41.4% of the participants were smokers. 47.7% had other general chronic illnesses. The highest level of degree was lower secondary education (57.7%), followed by high school diploma (22.2%), and university degree (15.6%). 4.4% did not finish or attend school at all. The limited ability to eat bothered patients most during their stay at the hospital.

## Discussion

It is important for medical and dental professionals to evaluate a patient’s OHRQoL in order to meet patient’s needs, plan appropriate care, and monitor the treatment process [20, 21].

The validity, sensitivity, and specificity of OHIP-49 as a measuring instrument for OHRQoL were validated in a huge variety of settings [4, 21]. Self-completion of the questionnaire has proven suitable for administration of the OHIP-49 [22, 23].

In 2003 John et al. randomly surveyed 2050 German citizens and created a reference group for OHRQoL OHIP-G scores [19]. In comparison, patients with fascial space abscesses scored significantly higher than the unaffected population. Even at follow-up OHIP-G scores with a mean of 30.80 (for patients without dentures) and a 95% confidence interval of 19.07 and 42.53 patients were still distinctly higher than the general German population without dentures. According to John et al., patients wearing dentures (removable or complete) generally seem to score higher. In this study patients with fascial space abscesses wearing dentures initially reached lower OHIP-G scores than patients without dentures, at follow-up edentulous patients scored higher. Furthermore, the gap between baseline and follow-up scores was noticeably smaller in patients wearing dentures than in patients without dentures. This might be due to some kind of tolerance that patients wearing dentures towards oral pain and indisposition have developed. This tolerance might reduce the impact the acute event has on their OHRQoL. An early and suitable prosthetic rehabilitation might alleviate the impact on OHRQoL [24].

In general, higher OHIP-G scores of patients with fascial space abscesses might have also been due to the acute emergency situation patients found themselves in. Acute illnesses have poorly been evaluated in OHRQoL research. Chronic diseases such as chronic obstructive pulmonary disease, Parkinson’s disease or TMJ seem to be more suitable for assessments of OHRQoL [10, 25]. Nevertheless, acute illnesses can affect long-term QoL as well. The impact of acute dental pain on OHRQoL has been evaluated in Croatia and Canada with results of impacted OHRQoL [26, 27]. Shueb et al. [10] compared OHRQoL in patients with acute dental pain to OHRQoL of patients suffering from TMJ, trigeminal neuralgia and persistent dentoalveolar pain. All study groups showed similar OHIP Scores [10]. In this study OHRQoL is lower in patients with fascial space abscesses than OHRQoL of the unaffected population. Unfortunately, there is no option to evaluate whether the illness itself or the impact of surgical treatment or hospitalisation negatively affects OHRQoL. Therefore, a recall period of 1 month was chosen in order not to only concentrate on the specific emergency situation at baseline evaluation. Furthermore OHIP-G scores were high not only at the acute incident but also after 3–6 months. After 3–6 months the acute emergency situation is over and daily routines have reestablished giving an insight to long-term psychological distress. This not only has an effect on patients themselves but on the health care system as a whole since low quality of life is known to promote illness [28]. “Social disability” is the only subsection that has not significantly changed at follow-up (Table 1). While a follow-up of 3–6 months has proven suitable for QoL follow-up measurements [29] it might have been too short for the “social disability” section to change, which includes psychological rather than oral items.

**Table 1:**
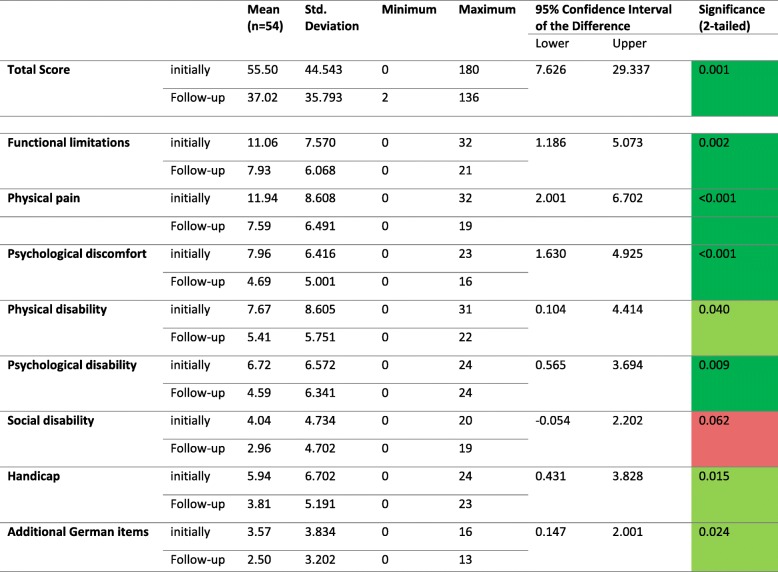
OHIP-G results and paired differences

OHIP-G scores for patients with fascial space abscesses in comparison. Light green = significant difference (*p* = < 0.05), dark green = highly significant difference (*p* = < 0.01), red = no significant difference

Although odontogenic fascial space abscesses are fairly common, it turned out to be extraordinarily difficult to find suitable patients to complete the questionnaires. While 121 patients met inclusion criteria, only 94 patients took part in the study and only 54 completed both OHIP-G questionnaires. The additional questions were repeatedly answered incompletely. This might have been due to a lack of motivation given the length of the questionnaire and the special fraction of patients asked to answer it. The OHIP itself contains 49 questions, the German version adds four items, and 16 additional questions were asked. The follow-up questionnaire was self-completed at home and had to be sent back by mail or e-mail. Because it was an effort to complete the questionnaire one might argue that only extremely reliable patients or patients who suffered psychologically completed both questionnaires, biasing the outcome. Slade et al. [30] reported similar difficulties when using the OHIP-49 considering the length of time for completion and the increased possibility of missing data. Therefore, in 1997, Slade et al. [30] and later on in 2012 van der Meulen et al. [31] introduced much shorter, valid versions of the OHIP-49. The OHIP-5 or OHIP-14 might have been a better choice to obtain more cases and a less biased outcome. Nevertheless, those shorter versions lack sensitivity in comparison to the OHIP-49 [10]. For a more reliable outcome further research has to include more numbers of cases and a broarder spectrum of patients.

Moreover, this study tried to detect underlying social reasons (such as habits, education, hygienic routines) for the development of dental fascial abscesses and reasons for the decreased OHRQOL with it (Table 2). Apart from well-known factors such as underlying general diseases, smoking, and social status, [28, 32, 33] it was interesting to see how patients subjectively evaluated their oral status. 46.3% found it to be “average”, 18.5% regarded their oral health status as “good”. This perception changes only slightly at follow-up. 33.3% don’t regularly do dental check-ups. A dentist is mainly consulted in case of dental pain. This might lead to an underestimation of oral health risks resulting for example in inflammation and abscess formation. 93% of patients plan to see a dentist more regularly in the future indicating the intention to prevent future similar events. A small percentage of patients did not regularly brush their teeth. While all of them used a toothbrush, some did not use toothpaste. Numbers do not considerably change at follow-up. According to our findings a fairly high percentage of patients might need better education in oral hygiene, and should be continuously motivated to attend regular dental check-ups. This could also improve long-term OHRQoL for patients making them more aware of future dental problems. After the loss of teeth suitable prosthetic rehabilitation has proven to increase the long-term OHRQoL in edentulous patients [24]. Although smoking is a well-known risk factor for gingivitis and plaque formation [32], the majority of patients in this study did not smoke. The advice to stop smoking should nevertheless be included in any therapeutic plan. The limited ability to eat bothered patients most during their stay at the hospital. This issue might be improved by better pain management and/or more enjoyable soft foods available at the hospital.

**Table 2:** Additional questions asked at baseline and at follow-up

Question	Initially		At 3–6 months follow-up	
	Frequency	%	Frequency	%
How many teeth do you have?	None (*n* = 1)	2.3	None (*n* = 3)	6.0
	1–9 teeth (*n* = 2)	4.5	1–9 teeth (*n* = 3)	6.0
	10–19 teeth (*n* = 11)	25	10–19 teeth (*n* = 14)	28.0
	20 or more teeth (*n* = 30)	68.2	20 or more teeth (*n* = 30)	60
How would you describe your current oral health status?	Bad (*n* = 10)	21.3	Bad (*n* = 10)	19.6
	Average (*n* = 25)	53.3	Average (*n* = 24)	47.1
	Good (*n* = 10)	21.3	Good (*n* = 15)	29.4
	Very good (*n* = 2)	4.3	Very good (*n* = 2)	3.9
How often do you brush your teeth?	Never (*n* = 1)	2.1	Never (*n* = 0)	0
	Once a month (*n* = 2)	4.3	Once a month (*n* = 1)	2.0
	Once a week (*n* = 2)	4.3	Once a week (*n* = 1)	2.0
	Once a day (*n* = 9)	19.1	Once a day (*n* = 14)	28.0
	At least twice a day (*n* = 33)	70.2	At least twice a day (*n* = 34)	68.0
Do you use a toothbrush for oral hygiene?	Toothbrush (*n* = 45)	97.8	Toothbrush (*n* = 50)	100
Do you use toothpaste when brushing your teeth?	Yes (*n* = 45)	95.7	Yes (*n* = 49)	98
	No (*n* = 2)	4.3	No (*n* = 1)	2
Are you wearing dentures?	Yes (*n* = 34)	27.7	Yes (*n* = 34)	32
	No (*n* = 13)	72.3	No (*n* = 16)	68
Do you smoke?	Yes (*n* = 19)	41.4	Yes (*n* = 19)	62.7
	No (*n* = 28)	59.6	No (*n* = 32)	37.3
What was the reason for your last dental consultation?	Check-up (*n* = 10)	21.3	Check-up (*n* = 10)	22.5
	Pain (*n* = 26)	55.3	Pain (*n* = 26)	52.5
	Concrete treatment (*n* = 7)	14.9	Concrete treatment (*n* = 11)	22.5
	I cannot remember (*n* = 4)	8.5	I cannot remember (*n* = 2)	4.5
How long ago was your last dental check-up?	Over 6 months ago (*n* = 27)	57.4	Not asked at follow-up	
	6–12 months ago (*n* = 71)	14.9		
	More than 1 year but less than 2 years ago (*n* = 3)	6.4		
	More than 2 years but less that 5 years ago (*n* = 4)	8.5		
	More than 5 years ago (*n* = 3)	6.4		
	I cannot remember (*n* = 3)	6.4		
How often do you do dental check-ups?	Every 6 months (*n* = 14)	29.8	Not asked at follow-up	
	Every 12 months (*n* = 12)	25.5		
	Every 2 years (*n* = 3)	6.4		
	No regular check-ups (*n* = 18)	38.3		
Why did you mainly consult our clinic?	Extensive swelling (*n* = 33)	73.3	Not asked at follow-up	
	Trouble while opening the mouth (*n* = 9)	20.0		
	Difficulties in swallowing (*n* = 1)	2.2		
	Distress in breathing (*n* = 1)	2.2		
	Overall feeling of illness (*n* = 1)	2.2		
Do you plan to regularly see a dentist in the future?	Yes (*n* = 42)	93.3	Not asked at follow-up	
	No (*n* = 3)	6.7		
Do you suffer from any general illnesses?	Yes (*n* = 21)	47.7	Not asked at follow-up	
	No (*n* = 23)	52.3		
What is your highest level of education?	Did not finish or attend school (*n* = 2)	4.4	Not asked at follow-up	
	Lower secondary education (*n* = 26)	57.7		
	High school diploma (*n* = 10)	22.2		
	University degree (*n* = 7)	15.6		
What bothers you most during your hospitalisation?	Difficulties in swallowing (*n* = 8)	17.8	Not asked at follow-up	
	Limited ability to eat (*n* = 14)	31.1		
	Difficulties sleeping (*n* = 8)	17.8		
	Pain (*n* = 7)	15.6		
	Other (*n* = 8)	17.8		

Analysis of the additional questions asked at baseline and follow up, comparing frequencies and percentages

OHRQoL creates, next to clinical assessments, a second dimension to the valuation of a patient’s oral status [19]. In order to provide best medical care, even in acute medical conditions therapeutic decisions should involve a patient’s subjective suffering as well. Although sometimes a certain treatment such as incision and drainage of abscesses is inevitable, surrounding conditions such as pain management, number of days of hospitalisation, a patient’s ability to recover at home, psychological consultation, etc. should be taken into account for further therapeutic planning. Mücke et al. [34] showed in 2015 that when it comes to fascial space abscesses early incisions under local anesthesia should be performed rather than waiting for operation room capacity. Incisions under general anesthesia should follow [34]. This might also affect OHRQoL, taking into account that a patient might wait in pain and distress for hours for incision and drainage under general anesthesia, when an early intraoral incision could have enhanced suffering and reduced psychological trauma. For 73.3% of patients the main reason for emergency admittance was extensive swelling, which might already have been reduced by an early incision in local anesthesia.

## Conclusions

Results suggest that the OHRQoL is significantly lower in patients with fascial space abscesses than the unaffected population, evaluated by John et al. in 2003, not only at the acute incident but also after 3–6 months [19]. This affects not only patients themselves but the health care system as well. Therapy of acute conditions should try to follow holistic approaches as well as it does for chronic diseases. Psychological factors should be taken into consideration when it comes to potentially life threatening acute conditions, and psychological assistance may be offered if necessary. Furthermore, patients should be educated better in terms of oral hygiene routines and may be introduced to a full dental hygiene program.

## Data Availability

The datasets used and/or analysed during the current study are available from the corresponding author on reasonable request.

## Abbreviations

MRONJ: Medication-Related Osteonecrosis of the Jaw
OHIP: Oral Health Impact Profile
OHIP-G: German Version of the Oral Health Impact Profile
OHRQoL: Oral Health-Related Quality of Life
QoL: Quality of Life
TMJ: Temporomandibular joint disorders
